# Gunshot Injuries of the Lungs

**Published:** 1918-08

**Authors:** 


***Gunshot Injuries of the Lungs.*** By Capt. EGGERS, *Surgery, Gynecology and Obstetric*, June, 1918.

This report is based on 30 cases studied in 1916 in a hospital at Eylau, Germany, and is of particular interest as it shows what the Central Powers are doing for these injuries. Sauerbruch reported 836 lung injuries in 22,145 wounded. Of 300 dead soldiers examined on the field 30 o/o had lung injuries. He believes that the mortality of injuries of the thorax is somewhat over 40 o/o.

The author summarizes the treatment of gunshot wounds of the lung as follows :

1. Treatment of clean, smooth wounds of the thorax and lungs is simple and strictly conservative. Rest in bed, elevation of the upper part of the body, morphine, dry sterile dressing, no transportation.

2. Perforating gunshot wounds of the thorax and lungs with a closed pneumothorax or without one, should be treated conservatively.

3. Hemothorax producing alarming symptoms of compression should be aspirated early, removing just enough fluid at first to relieve the symptoms.

4. Hemothorax running a normal course but showing no or little tendency to absorption should be aspirated to prevent the formation of a thickened pleura, contraction of the lung, etc

5. An infected hemothorax should either be aspirated at first and later have a rib resection, or if the symptoms are urgent, the resection should be done at once.

6. An open pneumothorax with a small external opening should be closed by suture if the wound is clean, otherwise by a fir, a dressing, or a tampon.

7. A pneumothorax with a large opening should promptly be treated surgically. If only the thoracic wall is injured the wound edges should be excised and the lung sutured into this window. In case the lung also has been perforated, this wound should likewise be excised and sutured, and this portion of the lung then fastened into the thoracic window (Sauerbruch, Landois, Burckhardt, Jehn).

8. In order to do these operations satisfactorily, it is advisable to have a simple positive pressure apparatus on hand.

9. In perforating injuries of chest and abdomen by rifle bullet or shrapnel balls, in which the course of the missile would indicate that the stomach or intestines might be injured, a primary laparotomy should be done at once. The thorax wound is treated conservatively. If the injury is due to shell fragment and an open pneumothorax is present, it is better to do a trans-diaphragmatic laparotomy. Injury to the abdominal organs is attended to through the opening in the diaphragm.

